# Making Better Use of the Policies and Funding We Already Have

**Published:** 2010-08-15

**Authors:** Raymond J Baxter

**Affiliations:** Community Benefit, Research and Health Policy, Kaiser Foundation Health Plan, Inc

## Abstract

The potential for population health reform could be enhanced by assessing whether we have made the most of policies and resources already available. Opportunities to promote population health independent of major changes in resources or public authority include the following: enforcing laws already in effect; clarifying and updating the application of long-standing policies; leveraging government's and the private sector's purchasing and investment clout; facilitating access to programs by everyone who is eligible for them; evaluating the effectiveness of population health programs, agencies, and policies; and intervening to stop agencies and policies from operating at cross-purposes.

## Optimizing Existing Resources to Improve Population Health

Proposals to improve the health of Americans typically rely on substantive changes in public policy, additional dedicated resources, or both. For example, some public health leaders have proposed dedicated funding for a "wellness trust." Accomplishing large-scale changes in law, regulation, and funding usually requires mobilization and negotiation among powerful interests and competing priorities, often with uncertain outcomes. Moreover, economic downturn makes resources scarce, and political partisanship makes consensus remote.

Population health policy reformers could also assess whether government agencies have made full and intended use of the policies already in effect and the resources already available. While the chronic underfunding of population health in the United States calls for new policies and programs funded with new resources, better implementation of existing policy may not require new resources. Officials, interest and advocacy groups, and the media need to understand the extent to which public agencies have executed current policies, optimized the use of available resources, and learned from rigorous evaluation using the best available methods. Government also needs to make the most of the authority it already has, better direct the private resources at hand, and get more performance out of current policy assets.

Such assessment, rigorously conducted, is likely to find some work that, done better, would free up resources; activities that should be stopped; and programs that require more investment. Moreover, a proper assessment would suggest how additional resources could be used in ways that multiply benefits. A tobacco tax, for example, deters consumption and can simultaneously fund prevention. Obesity prevention advocates now support similar taxes on sweetened beverages to reduce use and fund nutrition programs (1,2).

Proven, funded measures exist that can enhance the health of Americans without new laws or with carefully targeted new funding. Enforcing and publicizing these measures is likely to make a difference. This is the thinking, for instance, behind states' "click it or ticket" campaigns to enforce long-standing seatbelt laws and social marketing around enforcement of drunk-driving laws. Similarly, parents are beginning to organize to ensure that schools provide their children the physical education that states already require.

Opportunities also exist to apply existing policies and funding streams in ways that could be more effective. For instance, administrative changes in the US Department of Agriculture food stamp education program would allow states to use these dollars to support community environmental changes (now explicitly proscribed) (3), and administrative simplification could greatly facilitate the enrollment of children in the Children's Health Insurance Program (4).

Government and private organizations that have converging interests in improving health could undertake assessments that range more widely than those suggested in this article. Such assessments are needed to identify political and financing strategies that could reduce impediments to making better use of existing authority and funds. Many of these impediments are deeply rooted in the politics of interest groups and inter- and intra-governmental relationships. However, identifying these impediments systematically and devising ways to address them are outside the scope of this article. Moreover, some apparent opportunities to improve health that emerge from systematic assessment may not, on analysis, generate benefits that justify the political effort to achieve them. The purpose of what follows is to clarify the potential to improve population health by using existing policy and resources.

## Enforcing Existing Health-Promoting Laws and Regulations

Implementing policy that has already been enacted offers an opportunity to improve population health. For example, physical education and nutritional content of food in schools are covered in school wellness policies required by the Child Nutrition Act and by many state laws (5,6). Without local school champions and active parental involvement, good intentions often have been undercut by failed execution or compromised by competition for space in the school day for other subjects (7). Similarly, Medicaid requirements to provide preventive services for children are commonly ignored. The same is true for many environmental laws and regulations that affect air quality (8), smoking in public places (9), consumer protection with regard to toys and household items, and pedestrian and cyclist safety (10).

## Clarifying Expectations for "Community Benefit" From Nonprofit Hospitals

An opportunity to improve population health lies in how the Internal Revenue Service (IRS) and state attorneys general construe the "community benefit" provided by nonprofit hospitals and health plans as a condition of their tax-exempt status. Historically, these community benefits (estimated at $30 billion annually nationwide) have been poorly defined and inconsistently reported and quantified (11). Three schools of thought have dominated. A traditional regulatory view equates community benefit narrowly with "charity care" (free or discounted episodic care, usually in hospital emergency departments) for low-income patients who cannot pay some or all of the cost. A mainstream perspective in provider organizations counts charity care plus research, health professionals' education, and losses on underreimbursed public programs such as Medicaid. A population health perspective, in contrast, views community benefit as a wide array of community health improvement activities, determined by assessing local health needs (12).

Recently, IRS described 2 categories of activities that may be reported on IRS Form 990 for tax-exempt organizations: "community benefit," comprising the regulatory and provider perspectives described above, and "community building," which includes many of the programs considered community benefit from the population health perspective. IRS intends to analyze 2 years of reporting results before finalizing its requirements. Some population health advocates worry that IRS may determine that community health improvement activities do not count toward community benefit expenditure expectations. In that event, a traditional health fair (where uninsured people are screened free of charge for disease but not followed for treatment) might count, but a large-scale, multiyear, multisector community health initiative to reduce obesity might not qualify. In that case, nonprofit health organizations would have no incentive, other than a mission commitment, to pursue population health improvement initiatives. If IRS makes clear that comprehensive, community-based primary prevention activities and communitywide clinical improvement activities are included as community benefit — and if health care reform at the national or state level gradually reduces the need for traditional charity care — hundreds of millions, even billions, of dollars could become available for population health.

## Using the Purchasing and Investment Power of Government and the Private Sector

Another largely untapped resource is the considerable power that public entities have to improve health through their purchasing and investment practices. Many of these actions can be carried out by executive order or by management discretion.

Public agencies wield enormous purchasing power. Two opportunities involve healthy nutrition and environmentally responsible materials procurement. Government agencies can model and reward the purchase of healthy foods through the way they administer federal and state nutrition programs (the Special Supplemental Nutrition Program for Women, Infants, and Children; the Supplemental Nutrition Assistance Program, school meals) (13). Public organizations can directly promote health by selecting healthier foods for their vending machines and cafeterias; purchasing fresh, sustainably (and locally) farmed pesticide- and antibiotic-free foods; and labeling nutritional content (13). Public agencies can vigorously control the public purchase of supplies containing toxic materials with adverse health consequences, such as mercury, lead, bisphenol A, and polyvinyl chloride.

Similar health-promoting strategies could be incorporated into the investment policies of public pension and investment bodies. Investment standards can be disincentives to socially negative activities (production, sales, and marketing of tobacco and firearms, for example) or promote socially positive activities (economic development and green jobs) (14).

By promoting health through purchasing and investment strategies, government could reinforce and encourage the adoption of similar standards in the private sector. Private organizations, both for-profit and nonprofit, wield considerable purchasing and investment power, though it is not as concentrated as that of government. On the other hand, the private sector operates with fewer constraints than government. Many large companies, particularly those with global reach, already have adopted corporate social responsibility policies governing their environmental, employment, economic, and human rights impacts. They have formed trade associations and initiated partnerships with universities and nongovernmental organizations (such as Health Care Without Harm) to advance these policies and change purchasing practices. The changes adopted by such private companies, and in turn by their supply chains, extend all the way to original producers and to their employees and communities.

Moreover, investment practices of private and nonprofit organizations can also promote health. Nonprofits are a particular opportunity because many of them are funded in part by government and are sensitive to its goals, and others receive substantial funding from endowed foundations, many of which are making socially responsible investments. Although not as powerful as large public employee investment funds, their practices could still influence the prevailing sense of acceptable and appropriate investment policies. Evidence is mounting that socially responsible investment funds — those that screen out tobacco and firearms and sometimes alcohol and pornography — perform equivalently to general equity funds (15,16). More nonprofits are moving to invest proactively in community redevelopment and other activities that involve social determinants of health.

## Enrolling the "Eligible but Not Enrolled" Populations in Public Programs

"Eligible but not enrolled" identifies the millions of low-income people who are qualified for but not enrolled in public benefits, including health insurance, food and heating assistance, and social services. An estimated 25% of people who are eligible for Medicaid and the Children's Health Insurance Program (17) and 34% of those eligible for food stamps (18) are not enrolled in these programs.

Many federal and state dollars are unspent because of inadequate public management rather than political conflict or efforts to control spending or reduce fraud and abuse. Reasons include poor communications, stigma, and administrative barriers to enrollment. Public organizations and the private contractors they hire to administer programs erect such barriers as frequent requalification periods, lengthy application forms, complex documentation requirements, multiple in-person interviews, inaccessible venues for application, linguistic and cultural barriers, or lack of public information (19). Whatever their causes, these barriers often waste time and money in ways that can be calculated.

Food stamps, for example, are 100% federally funded (not counting a small state administrative cost) and generate $1.80 in economic activity for every dollar expended, yet only recently have states acted to facilitate enrollment (19). Similarly, electronic eligibility determination and application filing have expanded coverage for eligible people and lowered administrative costs (4,20).

## Increasing the Efficiency, Effectiveness, and Yield of Government Programs

Resources and authority are wasted when ineffective tactics are employed, interventions are poorly designed or targeted, agencies and policies work at cross-purposes, and evaluation of what works is not timely or well integrated into practice. Many public policies, moreover, undermine population health (for example, abstinence-only education, subsidizing commodity crops that contribute to obesity, and preventing disparagement of "bad food" as a condition for receiving US Department of Agriculture funds).

Interagency coordination to achieve mutual health goals is frequently recommended but infrequently practiced. A notable example of coordination is the California Strategic Growth Council, in which the state's agencies for health and human services, environmental protection, business and transportation, and natural resources coordinate their efforts related to sustainability and health-promoting changes to the built environment.

Broad-based general community planning offers additional opportunities to improve population health. Concepts such as health impact assessments of government policies and actions represent the European tradition of health in all policies. These concepts inform the new Healthy People 2020 goals for the nation. The public health planning groups established to help California implement its greenhouse gas emission standards also employ these concepts (21,22). Provisions in a small but increasing number of general plans and redevelopment district plans across the country promote health by increasing the walkability and bikeability of communities, improving air quality, and supporting more grocery stores and parks (23). Some communities have established joint-use agreements that link assets of different organizations, such as school athletic fields and county parks, that contribute to health.

## Using the Evidence to Design and Target Policies Effectively

Assessment of potential for making better use of existing policy and resources would benefit from more rigorous evidentiary standards for health interventions that affect populations. Large-scale community health interventions often have been criticized for lack of a scientific evidence base. Moreover, arguments among experts about appropriate methods for evaluating population health interventions have impeded use of the most persuasive contemporary tools of evaluation, especially systematic reviews that make careful use of both experimental and observational research designs. For example, systematic reviews conducted for the Centers for Disease Control and Prevention's *Guide to Community Preventive Services* have demonstrated the effectiveness (as well as the absence of evidence of effectiveness) of numerous public health measures (2,24,25). Another example is a systematic review by the Campbell Collaboration that found that the widely used Drug Abuse Resistance Education program is not effective, thus establishing an argument for reallocating funds. Similarly, the Institute of Medicine's Committee on an Evidence Framework for Obesity Prevention Decision-Making is developing recommendations that take account of the best evidence in an area in which advocacy sometimes has been ahead of science.

Incentive programs could be assessed for their contribution to population health. For instance, payment-for-performance schemes could reward improved performance in targeting clinical preventive services to reduce disparities that result from race and socioeconomic status.

## Practicing What You Preach: Government as Example

Public agencies' practices could be assessed to measure the extent to which they embrace risk reduction and harm reduction (such as eating healthy food, using clean needles, and encouraging condom use), openly acknowledge and address health issues (such as domestic violence and workplace safety), and reward behavior that contributes to health (such as economic development, public transit as a substitute for automobile use, environmental justice). By assessing its role in promoting health, government could set an example for private-sector organizations and nonprofits.

Indeed, private and nongovernmental organizations may more easily make health-promoting organizational changes that reflect the aims of public policy. Nongovernmental organizations have some latitude in deciding how to implement public policy — for example, prohibiting indoor smoking, sorting recyclables, providing ergonomic assessments for workers, subsidizing mass transit, or ensuring regular breaks and family leave. Moreover, nongovernmental organizations could also lead through voluntary practices — for instance, offering lactation spaces for breastfeeding mothers, stocking healthy food in vending machines, opening stairwells for regular use, providing bike stalls and showers, subsidizing gym memberships, or encouraging people to stay home when they are infectious. Such voluntary action could prompt public organizations to adopt similar measures.

## Conclusion: Get More out of What We've Got

The United States faces enormous population health challenges. Policy change and reallocation of public resources are essential to improve population health. Assessment is the first step in making existing policy and resource allocation more effective. Assessment, at every level of government, in nongovernmental organizations, and in communities, is necessary to select opportunities to improve population health and then devise political and reallocation strategies to attain them.
